# Preparation and Microstructure of High-Activity Spherical TaNbTiZr Refractory High-Entropy Alloy Powders

**DOI:** 10.3390/ma16020791

**Published:** 2023-01-13

**Authors:** Shenghan Gao, Ao Fu, Zhonghao Xie, Tao Liao, Yuankui Cao, Bin Liu

**Affiliations:** State Key Laboratory of Powder Metallurgy, Central South University, Changsha 410083, China

**Keywords:** refractory high-entropy alloy, TaNbTiZr, spherical powder, EIGA, PREP

## Abstract

High-activity spherical TaNbTiZr refractory high-entropy alloy (REHA) powders were successfully prepared by electrode induction melting gas atomization (EIGA) and plasma rotating electrode process (PREP) methods. Both the EIGAed and PREPed TaNbTiZr RHEA powders have a single-phase body-centered cubic (BCC) structure and low oxygen content. Compared with the EIGAed powders, the PREPed powders exhibit higher sphericity and smoother surface, but larger particle size. The average particle sizes of the EIGAed and PREPed powders are 51.8 and 65.9 μm, respectively. In addition, both the coarse EIGAed and PREPed powders have dendritic structure, and the dendrite size of the EIGAed powders is larger than that of the PREPed powders. Theoretical calculation indicates that the cooling rate of the PREPed powders is one order of magnitude higher than that of the EIGAed powders during the solidification process, and the dendritic structure has more time to grow during EIGA, which is the main reason for the coarser dendrite size of the EIGAed powders.

## 1. Introduction

High-entropy alloys (HEAs), especially the refractory high entropy alloys (RHEAs), have been attracting tremendous attention due to their attractive mechanical properties [1,2,3,4,5]. RHEAs usually consist of four or more refractory metallic elements, such as W, Mo, Ta, Nb, Hf, Ti, Zr and V, etc., which endow the alloy system outstanding high-temperature properties, including high melting point [6,7,8,9], high strength and hardness [10,11,12], good oxidation resistance [13], and outstanding thermal stability [14]. For example, WNbMoTa RHEA with a single-phase body-centered cubic (BCC) structure has an ultra-high yield strength of 405 MPa at 1600 °C [9,10], which makes it possible to replace the widely used Ni-based superalloys and be used as the next-generation high-temperature materials. However, the RHEAs containing W and Mo elements generally show high room-temperature brittleness and densities (>13 g/cm^3^ [9]). The TaNbTiZr RHEA developed recently by replacing W and Mo with Ti and Zr can overcome the brittle and heavy bottlenecks and exhibits low density (8.9 g/cm^3^ [15]), as well as high yield strength (410 MPa at 1000 °C [16]) and fracture strain (>48% at room temperature [17]), which has attracted extensive interests.

However, the high melting temperature of RHEAs will inevitably bring challenges for traditional manufacturing technologies, e.g., casting, extrusion and forging to produce high-quality complex components. Additive manufacturing (AM) is an emerging direct forming technology capable of producing complex components that can effectively solve the troubles of poor formability and extend the applications of RHEAs. Spherical powders are the raw material for AM, and the sphericity is a key parameter affecting product quality [18,19]. Although extensive efforts have shown that the preparation of RHEAs can be achieved by AM of elemental powders, the AMed products have severe compositional segregation and unstable mechanical properties due to the large differences in the melting points of the employed elements [20,21]. The use of pre-alloyed spherical powders with a targeted composition can eliminate the above problems, thereby the preparation of the pre-alloyed powders is extremely important.

At present, inductively coupled thermal plasma spheroidization method is the main method for preparing spherical RHEA pre-alloyed powders. For example, Xia et al. [22] prepared the WTaMoNbZr RHEA powders by vacuum electron beam melting (VEBM), hydrogenation, and disk-milled and plasma spheroidization. The spheroidized powders have good sphericity and homogeneous elemental distribution, but a high oxygen content of 1678 ppm. Na et al. [23] also fabricated the spherical TaNbHfZrTi RHEA powders through vacuum arc melting, hydrogenation, jaw crushing, and ball-milling and plasma spheroidization, and the oxygen content still reaches 1650 ppm. The above studies show that although the plasma spheroidization method can achieve the preparation of spherical RHEA powders, however, the preparation process contains a complex hydrogenation-dehydrogenation reaction involving crucible melting and mechanical crushing, which can easily lead to the introduction of impurity elements, especially the oxygen.

Hence, how to realize the preparation of high-activity RHEA powders with high sphericity and purity is still a difficult problem to solve up to now. In this work, we chose the methods of cold crucible levitation melting (CCLM) combined with electrode induction melting gas atomization (EIGA) and plasma rotating electrode process (PREP), respectively, to prepare spherical TaNbTiZr RHEA powders. Since the preparation process is simple and all of the above preparation methods belong to crucible-free fabrication technology [24,25], the oxygen content of the achieved powders is low. More importantly, industrial-scale production of more than 100 kg of powder has been obtained by both the EIGA and PREP methods. The detailed characterization of the powders and the influence of the cooling rate during the solidification process on the microstructure of the powders were investigated.

## 2. Materials and Methods

**Figure 1:**
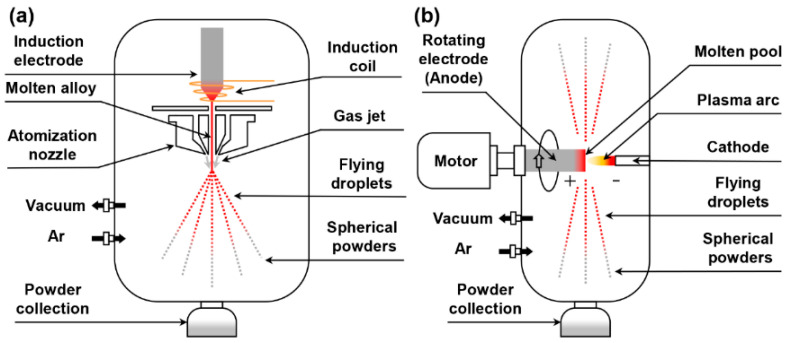
Schematic diagrams of the (**a**) EIGA and (**b**) PREP systems.

The TaNbTiZr RHEA ingots were fabricated from pure metals (purity > 99%) by CCLM and subsequently cast into the dimensions of Φ50 × 300 mm. Then, these bars were atomized into spherical powders via the EIGA and PREP methods, respectively, under high-purity argon atmosphere. Figure 1 shows the schematic diagrams of the EIGA and PREP systems. The EIGA method uses a tightly coupled nozzle to atomize the molten alloy into fine droplets by a high-pressure airflow, while the PREP method relies on the centrifugal force of the electrode rotating at high speed to crush the molten alloy into fine droplets [26,27,28]. The droplets cool down rapidly under an argon atmosphere and eventually form pre-alloyed powders. The vacuum before the argon purge in the EIGA and PREP devices is lower than 5 Pa and 10 Pa, respectively. The atomization pressure in the EIGA device is 3.8 MPa, and the electrode rotation speed in the PREP device is 20,000 rpm. Subsequently, both the EIGAed and PREPed powders with the particle size less than 105 μm were sieved for further microstructural characterization.

Metallic element contents were measured by an inductively coupled plasma mass spectroscopy (ICP-MS). Oxygen/hydrogen/nitrogen and carbon contents were determined by an O/N/H analyzer (LECO, TCH-600) and a C/S analyzer (LECO, CS-600), respectively. Powder size distribution was analyzed by a laser diffraction particle size analyzer (Malvern, Micro-plus). Phase constitution was detected by an X-ray diffractometer (XRD, Bruker D8) using Cu Kα radiation. Microstructural characterization was investigated by a scanning electron microscopy (SEM, FEI Nova Nano230) equipped with an electron-backscattered diffraction (EBSD) analyzer. Chemical analysis at the macroscale was performed by an Electron Probe Micro-analyzer (EPMA, JXA-8530F).

## 3. Results

### 3.1. Characterization of the TaNbTiZr RHEA Bars

**Figure 2:**
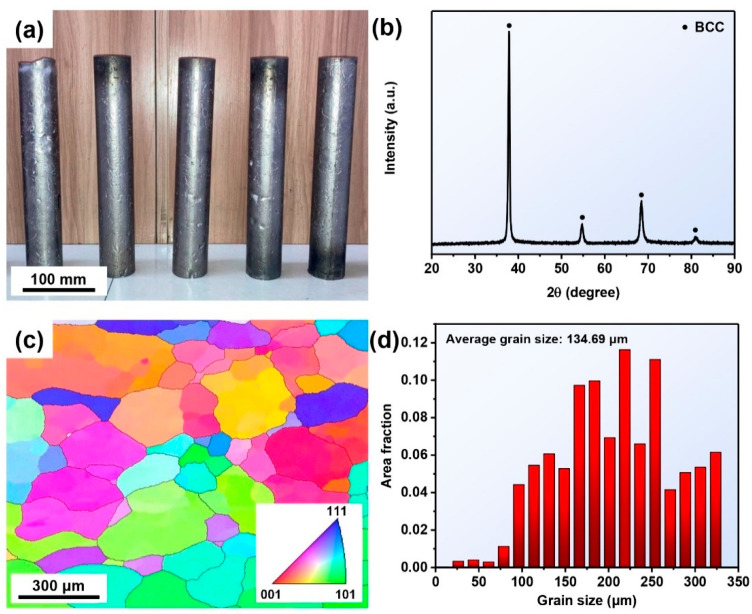
(**a**) TaNbTiZr RHEA bars; (**b**) XRD pattern, (**c**) EBSD IPF map, and (**d**) grain size distribution.

**Figure 3:**
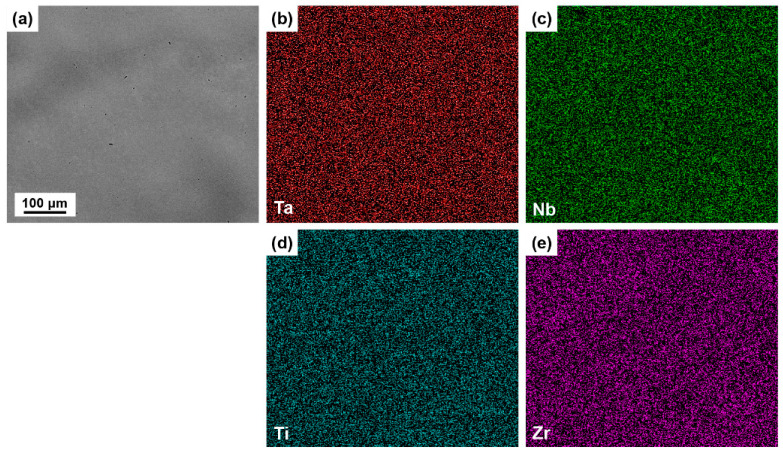
(**a**) SEM image of the TaNbTiZr RHEA bars; EDS mappings of (**b**) Ta, (**c**) Nb, (**d**) Ti, and (**e**) Zr, respectively.

The TaNbTiZr RHEA bars with 50 mm in diameter and 300 mm in length were prepared by CCLM, as shown in Figure 2a. The XRD pattern of the TaNbTiZr RHEA is given in Figure 2b, showing a single-phase BCC structure. The microstructure of the TaNbTiZr RHEA is displayed in Figure 2c, and the inverse pole figure (IPF) map reveals that the TaNbTiZr RHEA consists of equiaxial grains with a random grain-orientation distribution. The grain size distribution is summarized in Figure 2d, and the average grain size is nearly 134.69 μm. The oxygen content of the TaNbTiZr RHEA bars is ~488 ppm. To further investigate the composition uniformity of the TaNbTiZr RHEA bars, Figure 3 demonstrates the back-scattered electron (BSE) image of the TaNbTiZr RHEA and the corresponding elemental distribution maps of Ta, Nb, Ti, and Zr. It can be clearly seen that the elemental segregation is very slight, and no detectable secondary phases exist, which indicates that the TaNbTiZr RHEA bars prepared by CCLM have good compositional homogeneity. This provides a strong guarantee for the subsequent preparation of powders with uniform composition and low oxygen content.

### 3.2. Characterization of the TaNbTiZr RHEA Powders

**Figure 4:**
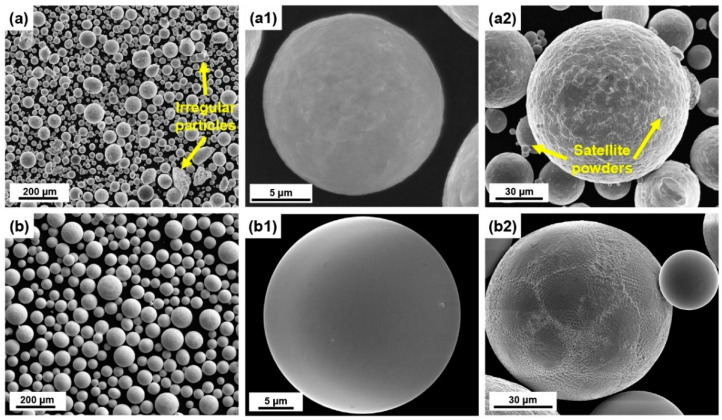
SEM images of the spherical TaNbTiZr RHEA powders at different magnifications: (**a**,**a1**,**a2**) the EIGAed powders and (**b**,**b1**,**b2**) the PREPed powders.

**Figure 5:**
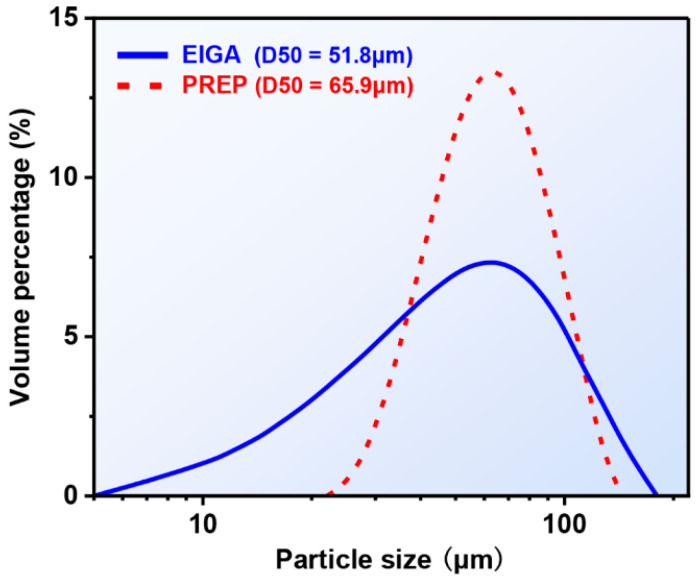
Particle size distributions of the EIGAed and PREPed TaNbTiZr RHEA powders.

**Figure 6:**
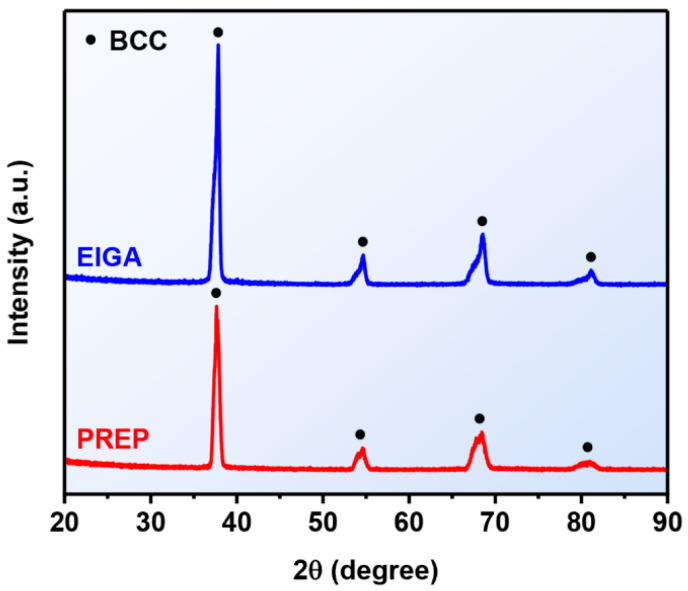
XRD patterns of the EIGAed and PREPed TaNbTiZr RHEA powders.

SEM images of the EIAGed and PREPed TaNbTiZr RHEA powders are shown in Figure 4. It can be seen that the EIGAed powders (Figure 4a) are nearly spherical in shape, but there are also some irregular shaped particles and satellite powders. The reason for the formation of satellite powders is mainly due to the fact that during gas atomization, the small molten droplets have a faster cooling rate and flying speed, which can easily collide with large molten droplets and solidify around them to form satellite powders [29]. The particle size distribution of the EIGAed powders is plotted in Figure 5, showing a typical Gaussian distribution. The particle size of the EIGA powder is mainly in the range of 30 to 100 μm, with a mean particle size (D50) of 51.8 μm. The high-magnified SEM images represented in Figure 4a1,a2 show that the fine powders have a smooth surface, while the surface of the coarse powders is rough due to the appearance of the dendritic structure. Compared with the EIGAed powders, the PREPed powders exhibit higher sphericity and smoother surface (Figure 4b). The PREPed powders also have a narrower size distribution with a D50 of 65.9 μm. Figure 4b1,b2 represent the SEM images of the PREPed powders at higher magnification, where the surfaces of the PREPed powders are smoother than the EIGAed powder at similar particle size. In addition, XRD analysis performed on the EIGAed and PREPed powders are shown in Figure 6, and all the powders keep a single-phase BCC structure from the as-cast state, indicating that no secondary phase formed during the preparation of powders.

The chemical composition of the EIGAed and PREPed TaNbTiZr RHEA powders are listed in Table 1. It can be seen that the actual compositions of the EIGAed and PREPed powders are in good agreement with the nominal composition, indicating that the powders fabricated by these two methods have good composition uniformity. In addition, the contents of impurity elements, especially the oxygen, are low. The oxygen contents of the spherical TaNbTiZr RHEA powders prepared in this work and other similar spherical RHEA powders [22,23,30] are listed in Table 2. Obviously, the oxygen content in the EIGAed powders (845 ppm) and PREPed powders (777 ppm) is significantly lower than that of the spherical high-activity RHEA powders prepared by plasma spheroidization method (~1700 ppm).

**Figure 7:**
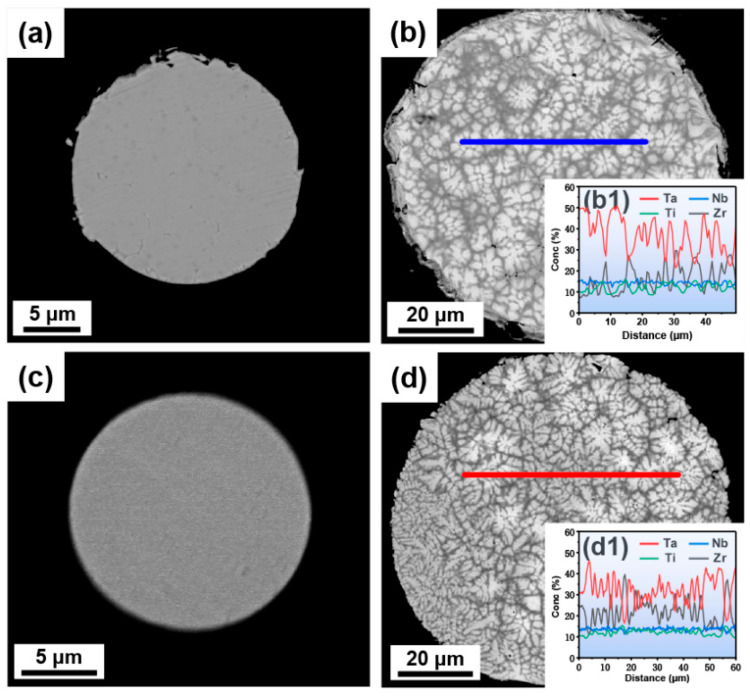
Cross-section microstructure of the (**a**,**b**) EIGAed and (**c**,**d**) PREPed TaNbTiZr RHEA powders; (**b1**) and (**d1**) are the elemental line scanning of the marked regions in (**b**) and (**d**), respectively.

**Figure 8:**
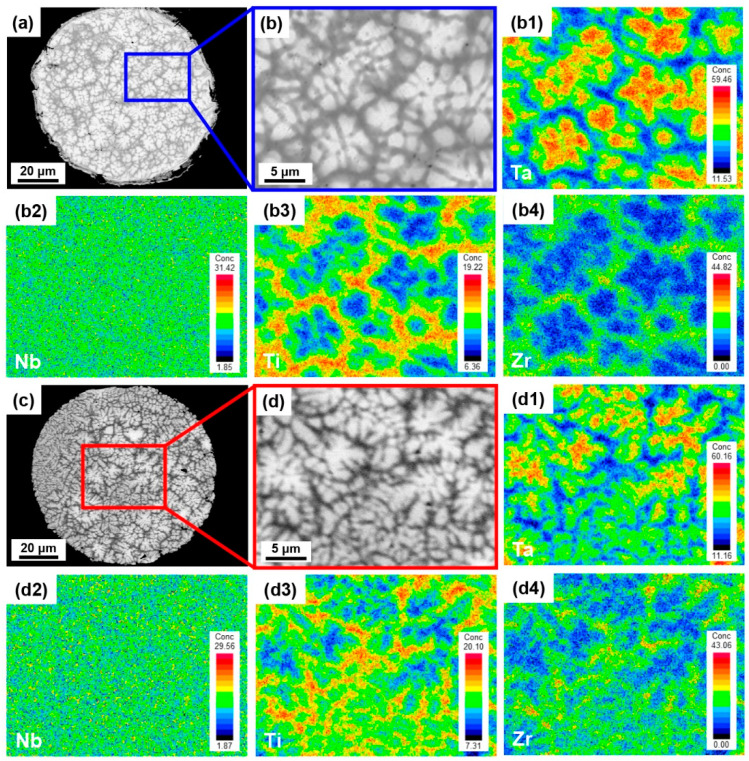
SEM images of the (**a**,**b**) EIGAed and (**c**,**d**) PREPed TaNbTiZr RHEA powders; (**b1**–**b4**) and (**d1**–**d4**) are the elemental mapping of the marked regions in (**a**) and (**c**), respectively.

The cross-sectional microstructure of the EIGAed and PREPed TaNbTiZr RHEA powders are shown in Figure 7. The fine powders and coarse powders exhibit different microstructure, i.e., the fine powders exhibit dendrite-free structure (Figure 7a,c), while the coarse powders have typical dendritic structure (Figure 7b,d). Moreover, it can also be seen that the dendritic structure of the EIGAed powders is coarser than that of the PREPed powders. The dendrite arm spacings in the EIGAed and PREPed powders are measured, and the corresponding values are 1.88 μm and 1.62 μm, respectively. The elemental line scannings in the coarse EIGAed and PREPed powders are shown in Figure 7b1,d1, respectively, and the curves of each element fluctuate obviously, indicating that there is elemental segregation in the dendritic structure. The composition fluctuation of the EIGAed powders is significantly larger than that of the PREPed powders, which means that the segregation in the EIGAed powders is more serious. The EMPA elemental mapping (Figure 8) was conducted to further investigate the elemental distribution of each element in the dendritic structure, and the EIGAed and PREPed powders show a similar elemental distribution tendency, that is, the Ta element with a higher melting point is enriched in the dendrite region, the Ti and Zr elements with lower melting point exhibit the opposite tendency, and the Nb element is evenly distributed in the dendritic and interdendritic regions.

## 4. Discussion

The microstructure of the powders is usually closely related to the cooling rate [31]. To analyze the effect of the cooling rate on the microstructure during the rapid solidification process, the cooling rate of spherical powders with various particle sizes under different preparation processes are calculated by numerical simulation.

For the EIGAed TaNbTiZr RHEA powders, the cooling rate *V_c_*_1_ of the powders can be calculated by the following formula [32,33]:

(1) $$V_{c\;1}=\frac{{6h}_{1}}{C_{d}\rho_{d}D}\left(T_{d}\;{-\;T}_{g}\right)$$

where *D* is the diameter of the TaNbTiZr RHEA droplets; *C_d_* is the theoretical specific heat capacity of the TaNbTiZr RHEA; *ρ_d_* is the theoretical density of the TaNbTiZr RHEA droplets, which is usually close to that of the solid state; *h*_1_ is the convection heat transfer coefficient; *T_d_* is the TaNbTiZr RHEA droplet temperature; *T_g_* is the argon temperature; and *h*_1_ can be expressed by the Ranz-Marshall relation [34]:

(2) $$h_{1}=\frac{k_{g}}{D}\left(2+0.6\sqrt{Re}\sqrt[{3}]{Pr}\right)$$

where *k_g_* is the argon thermal conductivity; ${Re=\rho }_{g}D\frac{u_{d}{-u}_{g}}{\mu_{g}}$ is the droplet Reynolds; $Pr=\frac{C_{pg}\mu_{g}}{k_{g}}$ is the argon Prandtl number; *ρ_g_* is the argon density; $u_{d}\;{-\;u}_{g}$ is the velocity difference between droplet and airflow; *μ_g_* is the argon dynamic viscosity; and *C_pg_* is the argon specific heat capacity per unit mass. When ignoring the speed difference between the high-speed argon and the atomized droplets flying during the atomization process [33], the *V_c1_* can be rewritten as:

(3) $$V_{c1\;}=\frac{{12k}_{g}}{C_{d}\rho_{d}D^{2}}\left(T_{d}-{\;T}_{g}\right)$$

For the PREPed TaNbTiZr RHEA powders, the cooling rate *V_c_*_2_ of the powders can be described as [31]:

(4) $$V_{c2}=\frac{{6h}_{2}}{C_{d}\rho_{d}D}\left(T_{d}-{\;T}_{g}\right)$$

where *h*_2_ is the heat transfer coefficient, and can be calculated by the following formula [31]:

(5) $$h_{2}=\frac{{2k}_{g}}{D}+0.6{\left(\frac{k_{g}^{4}\rho_{g}^{3}C_{g}^{2}}{y_{g}}\right)}^{\frac{1}{6}}{\left(\frac{v}{D}\right)}^{\frac{1}{2}}$$

where *C_g_* is the argon specific heat capacity; *y_g_* is the viscosity of argon; and *v* is the linear velocity of the rotating electrode edge, which is calculated to be 5.24 × 10^4^ mm s^−1^, since the electrode has a diameter *d* of 50 mm and rotation speed *r* is 20,000 rpm. Hence, the *V_c_*_2_ can be rewritten as:

(6) $$V_{c2}=\frac{{12k}_{g}}{C_{d}\rho_{d}D^{2}}\left(T_{d}-{\;T}_{g}\right)+\frac{3.6h_{2}}{C_{d}\rho_{d}}\left(T_{d\;}-{\;T}_{g}\right){\left(\frac{k_{g}^{4}\rho_{g}^{3}C_{g}^{2}}{y_{g}}\right)}^{\frac{1}{6}}{\left(v\right)}^{\frac{1}{2}}{\left(\frac{1}{D}\right)}^{\frac{3}{2}}$$

Table 3 summarizes the thermophysical constants of the TaNbTiZr RHEA and argon, and the preparation parameters. By substituting the above constants/parameters into Equations (3) and (6), respectively, the cooling rate of the EIGAed and PRERed powders can be expressed as:

(7) $$V_{c1}={4.68\;\times \;10}^{8}{\left(\frac{1}{D}\right)}^{2}$$

(8) $$V_{c2}={4.68\;\times \;10}^{8}{\left(\frac{1}{D}\right)}^{2}+{108.65\;\times \;10}^{6}{\left(\frac{1}{D}\right)}^{\frac{3}{2}}$$

According to the above formulas, the cooling rate of powders is negatively correlated with particle size during the solidification process. Meanwhile, for the powders with similar particle sizes, the cooling rate varies greatly under different preparation methods. For example, when the particle size is 18 μm, the cooling rates of the EIGAed and PREPed powders are 1.44 × 10^6^ and 2.87 × 10^6^ K/s, respectively; when the particle size is 80 μm, the cooling rates of the EIGAed and PREPed powders are 7.31 × 10^4^ and 2.25 × 10^5^ K/s, respectively. Obviously, for the same preparation method, the coarse powders have a longer solidification time than fine powders, so the dendritic structure is hard to form in the fine powders while the coarse powders have dendritic structure. For powders with the same particle size, the cooling rate of the PREPed powders is one order of magnitude higher than that of the EIGAed powders. The time for dendrite growth during PREP is significantly shorter than that in the EIGA process. Hence, the dendrite size in the PREPed powders is finer. Similar phenomena were also reported in the preparation of other pre-alloyed powders [35,36]. For example, He et al. [31] found that the microstructure of the PREPed high-Nb TiAl powders is closely related to the particle size, that is, coarse particles usually present dendritic structures, while finer particles exhibit featureless smooth structure.

## 5. Conclusions

In this study, novel high-activity spherical TaNbTiZr REHA powders were successfully prepared by EIGA and PREP methods. The phase composition and microstructure of the powders were characterized. The effect of cooling rate during the solidification process on microstructure evolution was investigated. The main conclusions are summarized as follows:

(1) Both the EIGAed and PREPed TaNbTiZr powders exhibit a single-phase BCC structure and have a low oxygen content (845 and 777 ppm for the EIGAed and PREPed powders, respectively).

(2) The PREPed powders show higher sphericity and smoother surface compared with the EIGAed powders. The average particle size (65.9 μm) of the PREPed powders is slightly larger than that of the EIGAed powders (51.8 μm).

(3) The dendritic structure appears in the coarse powders, and the dendrite size in the EIGAed powders is larger than that in the PREPed powders. The low cooling rate during EIGA is considered to be responsible for the larger dendrite size of the EIGAed powders.

(4) This work highlights the characters of TaNbTiZr RHEA powders prepared by EIGA and PREP method, but variable parameters is not studied. Future work focusing on the relation between processing parameters and powder properties is warranted.

## Figures and Tables

**Table 1:** Chemical composition of the EIGAed and PREPed TaNbTiZr RHEA powders.

Powder	Element	Ta (at%)	Nb (at%)	Ti (at%)	Zr (at%)	O (ppm)	N (ppm)	C (ppm)	H (ppm)
	Nominal	25.00	25.00	25.00	25.00				
EIGAed	Actual	25.12	24.14	25.48	25.26	845	124	150	12
PREPed	Actual	25.19	24.49	24.55	25.77	777	17	53	17

**Table 2:** Comparison of the oxygen content of the spherical TaNbTiZr RHEA powders with other similar RHEA powders prepared by different methods.

Alloys	Preparation Methods	O (ppm)	Ref
WTaMoNbZr	Melting + Hydrogenation + Crushing + Spheroidization	1678	[22]
TaNbHfZrTi	Melting + Hydrogenation + Dehydrogenation	1770	[30]
TaNbHfZrTi	Melting + Hydrogenation + Dehydrogenation + Spheroidization	1650	[23]
TaNbTiZr	CCLM + EIGA	845	This work
TaNbTiZr	CCLM + PREP	777	This work

**Table 3:** The thermophysical parameters of the TaNbTiZr RHEA and argon, and the corresponding preparation parameters [15,17,31].

Parameters	Symbol	Value
Specific heat capacity (TaNbTiZr)	*C_d_*	0.23 J g^−1^ K^−1^
Droplet density (TaNbTiZr)	*ρ_d_*	8.91 g cm^−3^
Droplet temperature	*T_d_*	2547.15 K
Argon temperature	*T_g_*	298.15 K
Argon thermal conductivity	*k_g_*	3.55 × 10^−2^ W m^−1^ K^−1^
Argon density	*ρ_g_*	9.7 × 10^−4^ g cm^−3^
Argon specific heat capacity per unit mass	*C_pg_*	5.21 × 10^−1^ J g^−1^ K^−1^
Argon viscosity	*y_g_*	4.62 × 10^−4^ g cm^−1^ s^−1^
Electrode diameter	*d*	50 mm
Electrode rotation speed	*r*	20,000 rpm

## Data Availability

The data presented in this study are available on request from the corresponding author.
